# Molecular determinants of response kinetics of mouse M1 intrinsically-photosensitive retinal ganglion cells

**DOI:** 10.1038/s41598-021-02832-9

**Published:** 2021-12-06

**Authors:** Yanghui Sheng, Lujing Chen, Xiaozhi Ren, Zheng Jiang, King-Wai Yau

**Affiliations:** 1 Solomon H. Snyder Department of Neuroscience, Johns Hopkins University School of Medicine, 725 North Wolfe St, Baltimore, MD 21205 USA; 2 Graduate Neuroscience Program, Johns Hopkins University School of Medicine, Baltimore, MD 21205 USA; 3 Present Address: Department of Neurobiology, Harvard Medical School, 220 Longwood Ave, Boston, MA 02115 USA; 4 Present Address: Vedere Bio II, Inc., 700 Main St, Cambridge, MA 02139 USA; 5 Present Address: Department of Ophthalmology, Baylor College of Medicine, 6565 Fannin St, Houston, TX 77030 USA

**Keywords:** Neuroscience, Cell signalling

## Abstract

Intrinsically-photosensitive retinal ganglion cells (ipRGCs) are non-rod/non-cone retinal photoreceptors expressing the visual pigment, melanopsin, to detect ambient irradiance for various non-image-forming visual functions. The M1-subtype, amongst the best studied, mediates primarily circadian photoentrainment and pupillary light reflex. Their intrinsic light responses are more prolonged than those of rods and cones even at the single-photon level, in accordance with the typically slower time course of non-image-forming vision. The short (OPN4S) and long (OPN4L) alternatively-spliced forms of melanopsin proteins are both present in M1-ipRGCs, but their functional difference is unclear. We have examined this point by genetically removing the *Opn4* gene (*Opn4*^*−/−*^) in mouse and re-expressing either OPN4S or OPN4L singly in *Opn4*^*−/−*^ mice by using adeno-associated virus, but found no obvious difference in their intrinsic dim-flash responses. Previous studies have indicated that two dominant slow steps in M1-ipRGC phototransduction dictate these cells’ intrinsic dim-flash-response kinetics, with time constants (τ_1_ and τ_2_) at room temperature of ~ 2 s and ~ 20 s, respectively. Here we found that melanopsin inactivation by phosphorylation or by β-arrestins may not be one of these two steps, because their genetic disruptions did not prolong the two time constants or affect the response waveform. Disruption of GAP (GTPase-Activating-Protein) activity on the effector enzyme, PLCβ4, in M1-ipRGC phototransduction to slow down G-protein deactivation also did not prolong the response decay, but caused its rising phase to become slightly sigmoidal by giving rise to a third time constant, τ_3_, of ~ 2 s (room temperature). This last observation suggests that GAP-mediated G-protein deactivation does partake in the flash-response termination, although normally with a time constant too short to be visible in the response waveform.

## Introduction

The mammalian retina mediates both image-forming and non-image-forming visual functions. Image-forming vision relies on light detection by rod and cone photoreceptors. It has high spatial and temporal resolution for sharply viewing an image and tracking its motion. Non-image-forming vision, on the other hand, is concerned primarily with subconscious photomodulation of whole-animal physiology and/or behavior, mediated mostly by the intrinsically photosensitive retinal ganglion cells (ipRGCs), which express melanopsin as their pigment^1–5^. IpRGCs comprise at least six distinct subtypes, M1 through M6, which differ in light sensitivity, morphology, pigment density, and projection targets in the brain^6–9^. M1-ipRGCs, which are the best-studied so far, are known to relay environmental irradiance information to the suprachiasmatic nucleus for circadian photoentrainment and to the olivary pretectal nucleus for the pupillary light reflex^6,9^.

In accordance with the primary function of rods and cones being to detect images and their associated motions, the rod/cone flash responses are fast and brief. In contrast, the intrinsic light response of ipRGCs serves primarily to signal ambient light versus darkness, or variations in light intensity, typically over a longer time scale than image-forming vision. Correspondingly, these flash responses of ipRGCs are slow and quite long-lasting, allowing integration of light signals over time. The waveform of the intrinsic M1-ipRGC’s dim-flash response can be described by the mathematical convolution of two single-exponential decays with average time constants (τ_1_ and τ_2_) of ~ 2 s and ~ 20 s, respectively, at room temperature^10–12^. This waveform is simpler than the dim-flash response of rods, which requires the convolution of four single-exponential decays for fitting^13^. The simple waveform of M1-ipRGCs’ intrinsic dim-flash response, however, belies the complexity of the underlying transduction events, in that τ_1_ and τ_2_ indicate only the dominant slow steps in phototransduction, with other considerably faster steps being invisible in the overall waveform. Phototransduction in M1-ipRGCs is now known to comprise the following sequence of events: melanopsin → Gα_q/11/14_ → phospholipase C-β4 (PLCβ4) → opening of TRPC6,7 channels, where “ → ” indicates activation^11,14,15^. However, it is still unknown which events correspond to the slow steps that dominate the shape of the dim-flash response.

Although the intrinsic dim-flash response of M1-ipRGCs has been well-studied^10^, it was thought at the time that all melanopsin molecules on an M1-cell are one and the same, producing a stereotyped light response. Subsequently, it became known that there are actually two alternatively-spliced protein isoforms, the short (OPN4S) and the long melanopsin (OPN4L), both present in M1-ipRGCs^16,17^. Thus, the question arises whether there are functional differences between these two isoforms, especially given that a specific ablation of OPN4S or OPN4L effected by RNAi led to differential effects on several non-image-forming visual behaviors in mice^18^.

In this work, we used mouse genetics to address the above questions. To check functional differences between OPN4S and OPN4L, we started with *Opn4*^*−/−*^ mice and re-expressed either OPN4S or OPN4L in them so that we could examine the light responses triggered by each melanopsin isoform separately. To investigate the kinetic correlates of different steps in M1-ipRGC phototransduction, we also genetically disrupted these steps individually and noted the consequences. As will be seen, although such an approach is very successful in understanding mouse rod phototransduction^19^, it has led to only very limited success in M1-ipRGCs, reflecting unusual complexity of these cells’ responses.

## Results

### Flash responses driven by OPN4S or OPN4L

Mouse OPN4S and OPN4L share the first 454 amino acids but differ in their C-terminal tails^17^. Quantitative RT-PCR indicates that *Opn4S* mRNA level is 40 times that of *Opn4L* in adult mice, and immunostaining with OPN4S- and OPN4L-specific antibodies indicates that both protein isoforms are present in each M1-ipRGC, although at unknown relative levels^17^. Because there are developmental changes in the mRNA levels of these two isoforms during the first month of age^16^, for simplicity we examined animals over one-month-old in all experiments.

**Figure 1:**
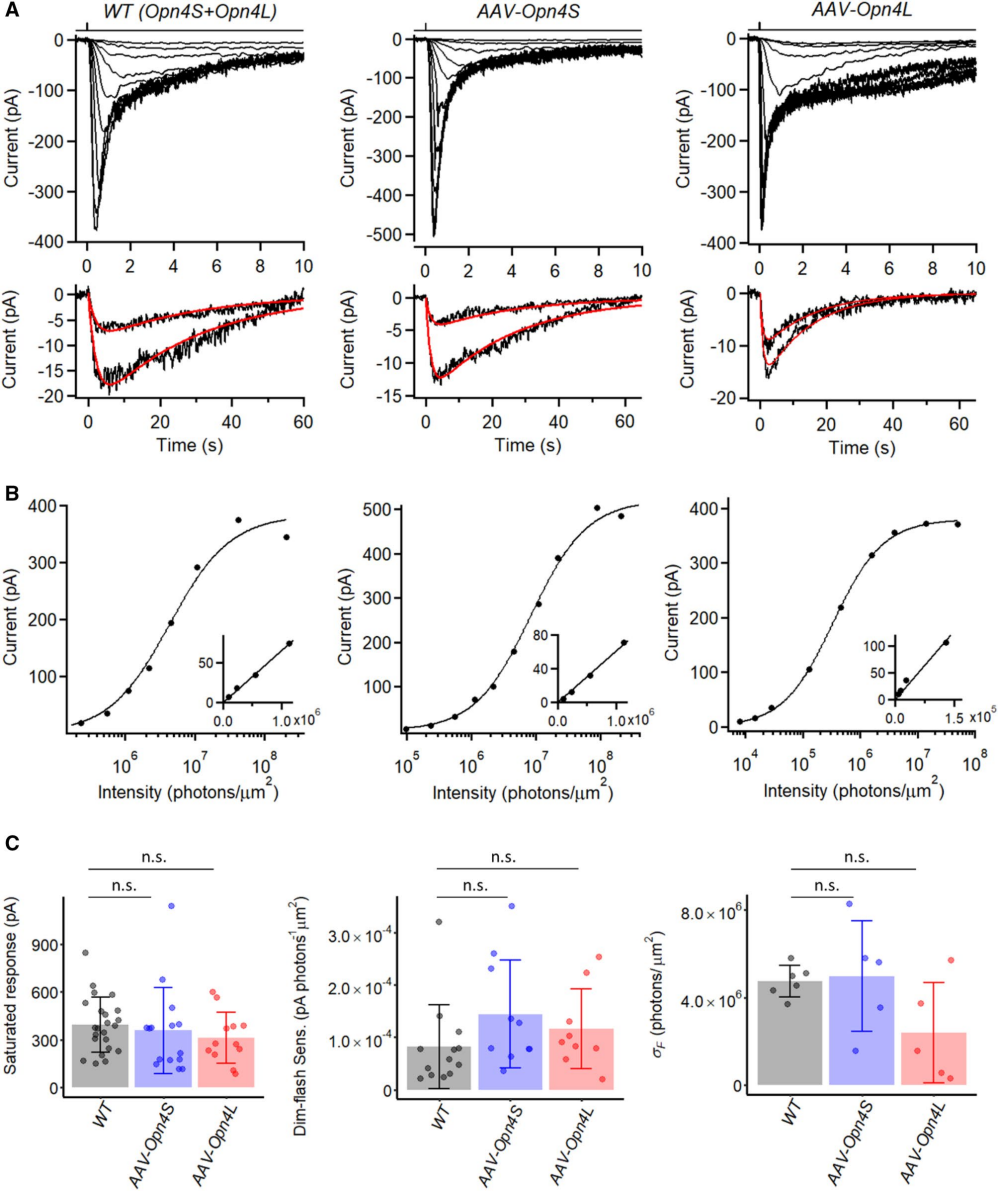
*Opn4*^*−/−*^ M1-ipRGCs expressing virally-delivered Opn4S and Opn4L isoforms show similar intrinsic light responses as WT M1-ipRGCs. (**A**) **Top:** Responses of a WT M1-ipRGC (left), an OPN4S-expressing M1-ipRGC (middle) and an OPN4L-expressing M1-ipRGC (right) to diffuse 50-ms flashes of varying intensities applied at time 0. Flashes were at 480 nm except for the two brightest flashes, which were white but converted to equivalent 480-nm light. **Bottom:** Two smallest dim-flash responses from **top**, fit with $A(e^{{ - t/\tau_{1} }} - e^{{ - t/\tau_{2} }} )$ and the same τ_1_ and τ_2_ values. WT: A = 9.7 and 24.0 pA, τ_1_ = 2.23 s, τ_2_ = 27.46 s. *AAV-Opn4S*: A = 21.8 and 41.4 pA, τ_1_ = 2.80 s, τ_2_ = 16.80 s. *AAV-Opn4L*: A = 11.0 and 17.8 pA, τ_1_ = 0.85 s, τ_2_ = 15.04 s. (**B**) Intensity-response relationships at transient peak plotted from (A), fit with Michaelis equation, $R = R_{max} I_{F} /\left( {I_{F} + \sigma_{F} } \right)$. WT: *R*_max_ = 383 pA and *σ*_*F*_ = 4.3 × 10^6^ photons µm^−2^. *AAV-Opn4S*: *R*_max_ = 521 pA and *σ*_*F*_ = 8.3 × 10^6^ photons µm^−2^. *AAV-Opn4L*: *R*_max_ = 378 pA and *σ*_*F*_ = 3.3 × 10^5^ photons µm^−2^. Insets: Foot of corresponding intensity-response relationship plotted on linear–linear scale. (**C**) Collected data on saturated response amplitude, dim-flash sensitivity, and *σ*_*F*_. Bars and error bars represent mean ± SD for each genotype. Because cells receiving diffuse full-field light stimuli may have large variations in illuminated area due to their different dendritic coverage, dim-flash sensitivity is shown only for cells stimulated with dim-flashes delivered via a 40-μm light spot centered on soma. Saturated response: 394 ± 172 pA for WT (n = 23), 358 ± 269 pA for *AAV-Opn4S* (n = 15), 312 ± 159 pA for *AAV-Opn4L* (n = 12); *p* = 0.36, Kruskal–Wallis test. Dim-flash sensitivity: 8.2 ± 8.0 × 10^–5^ pA photons^-1^ µm^2^ for WT (n = 13), 14.4 ± 10.3 × 10^–5^ pA photons^-1^ µm^2^ for *AAV-Opn4S* (n = 10), 11.6 ± 7.6 × 10^–5^ pA photons^-1^ µm^2^ for *AAV-Opn4L* (n = 9); *p* = 0.13, Kruskal–Wallis test. *σ*_*F*_: 4.8 ± 0.7 × 10^6^ photons µm^−2^ for WT (n = 6), 5.0 ± 2.5 × 10^6^ photons µm^−2^ for *AAV-Opn4S* (n = 5), 2.4 ± 2.3 × 10^6^ photons µm^−2^ for *AAV-Opn4L* (n = 5); *p* = 0.19, Kruskal–Wallis test. (n.s., not statistically significant).

We expressed OPN4S or OPN4L separately in the retinae of one-month-old *Opn4*^*−/−*^ mice (with *Opn4* replaced by the *tau-LacZ* marker gene, and crossed with a BAC-transgenic *Opn4*:*tdTomato* mouse line to label ipRGCs) by intravitreal injection of adeno-associated virus serotype 2 (*AAV2-hSyn-Opn4S/L-IRES-GFP-WPRE*; “Methods” section), with the IRES-GFP intended for identifying virus-infected cells (Fig. S1A). In about one month after injection, perforated patch-clamp recordings were made from infected *Opn4*^*−/−*^ M1-ipRGCs based on their strong tdTomato fluorescence, soma size (~ 10 µm in diameter), and co-localization with GFP fluorescence (Fig. S1B). As control animals, we used a BAC-transgenic *tdTomato* mouse line, which retains OPN4 and is labeled by tdTomato (see above). To elicit a flash-response family, we stimulated M1-ipRGCs with 50-ms, 480-nm flashes (*λ*_*max*_ for dark-adapted OPN4) with a large ~ 730-µm light spot centered on the recorded soma, such that it exceeded even the largest dendritic field of an M1-ipRGC. Figure 1A shows the flash-response family (top) together with the magnified dim-flash responses (bottom) from one cell of each genotype. The corresponding flash intensity-response relations measured at the responses’ transient peak are plotted in semi-logarithmic coordinates in Fig. 1B. The response behaviors of the three genotypes were broadly similar. With increasing flash intensity, the response time-to-peak (time lapse between flash application and transient peak of response) decreased progressively, which is a hallmark of light adaptation^13,20,21^. The intensity-response relation in each case can be fitted by the Michaelis equation, *R* = *R*_*max*_ [*I*_*F*_/(*I*_*F*_ + *σ*_*F*_)] (Ref.^10^), where *R* is response amplitude and *R*_*max*_ is saturated response amplitude at transient peak, *I*_*F*_ is flash intensity, and *σ*_*F*_ is the half-saturating flash intensity. The insets in Fig. 1B, plotted in linear–linear coordinates, indicate that the foot of each relation is linear, as expected from the fact that it takes only one absorbed photon to activate one melanopsin molecule and that the physical domain of phototransduction per active melanopsin molecule on the cell is spatially restricted, resulting in linear summation of the responses originating from separate domains at least when the number of photoexcited melanopsin molecules is sufficiently low to give no overlap between adjacent phototransduction domains^10^.

Figure 1C shows collected data of the saturated flash-response amplitude, absolute sensitivity and *σ*_*F*_ values for the three cohorts of cells. There was variation in the saturated response amplitude within each genotype, including WT. In principle, the expressions of OPN4S and OPN4L proteins in WT cells, being driven by the native *Opn4* promotor, did not have to be similar to those of the virally-expressed OPN4S and OPN4L, although in reality the average saturated response amplitudes across the three cell groups were quite similar (WT: 394 ± 172 pA, mean ± SD, n = 23; Opn4S: 358 ± 269 pA, n = 15; Opn4L: 317 ± 150 pA, n = 13). At least part of the variation could be due to cell-to-cell differences in dendritic-field sizes (thus light-collecting areas). Given the rapid increase followed by rapid decrease of the response at the initial transient, small variation in their relative rates could lead to a larger change in the amplitude of the transient peak. In this work, we focused on dim-flash responses and have not examined quantitatively the full waveforms of the saturated responses, especially during their prolonged decays (cf. Ref.^12^). The prolonged saturated-response decay of the OPN4L-expressing cell in Fig. 1A appears to be more dramatic than for the other two genotypes, but scrutiny of other cells did not reveal any consistency.

The dim-flash sensitivities were also broadly similar across the three genotypes, with their cohort averages being well within a factor of two of each other despite significant variations across individual cells of each cohort (Fig. 1C, middle). The *σ*_*F*_ parameter is basically the reciprocal of dim-flash sensitivity after discounting the contribution from the cell-to-cell variation in the saturated response amplitude when comparing across cells. This parameter, too, varied within a factor of two across genotypes (Fig. 1C, right). Given that the dim-flash sensitivities of OPN4S- and OPN4L-expressing cells were similar, it is not surprising that a combination of them, as represented by WT ipRGCs, also gave broadly the same sensitivity.

A recent detailed study of WT M1-ipRGCs has indicated that the various response parameters for M1-ipRGCs, including those described above, actually show considerable variations^12^, and what we observed between the two melanopsin isoforms were well within the ranges that this group reported. Because we are concerned here primarily with basic dim-flash-response properties associated with the isoforms, the response parameters chosen for our study are far from being comprehensive. We have not examined, for example, action-potential firings of the ipRGCs, or the effects of prolonged light, which require stable electrical recording for long periods.

**Figure 2:**
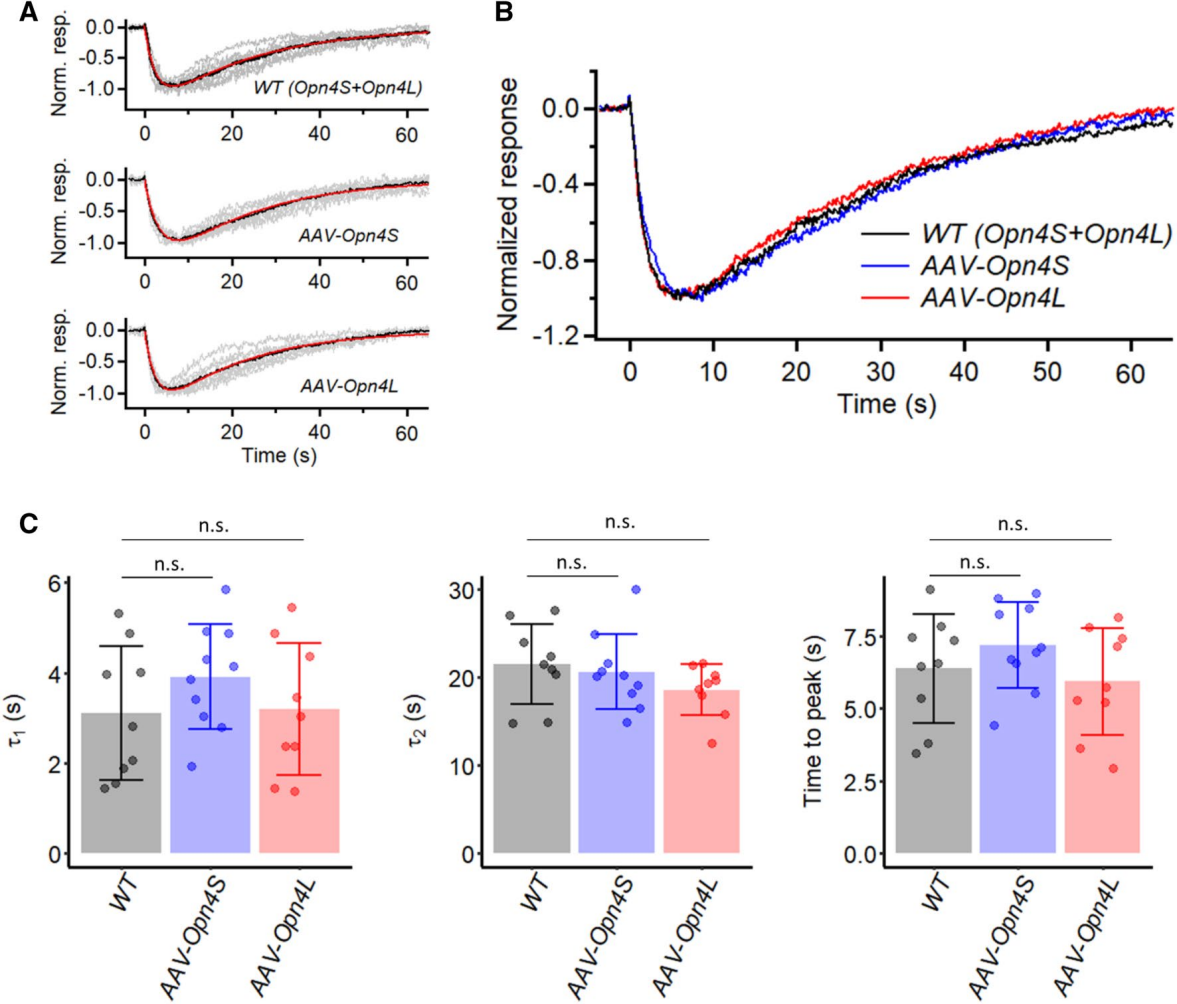
Kinetics of intrinsic dim-flash responses from *Opn4*^*−/−*^ M1-ipRGCs expressing virally-delivered Opn4S (*AAV-Opn4S*) or Opn4L (*AAV-Opn4L*), and from WT M1-ipRGCs which endogenously express both isoforms. All cells were stimulated via a light spot of 40-μm diameter centered on soma. (**A**) The averaged dim-flash responses (grey traces, 5–15 pA) recorded from nine WT M1-ipRGCs (top), ten *AAV-Opn4S* M1-ipRGCs (middle), and nine *AAV-Opn4L* M1-ipRGCs (bottom) are normalized in peak amplitude and superimposed. Averaged responses (black traces) are fit to the convolution of two single-exponential declines (red traces), $e^{{ - t/\tau_{1} }} *e^{{ - t/\tau_{2} }}$*.* WT: τ_1_ = 2.7 s, τ_2_ = 22.9 s. *AAV-Opn4S*: τ_1_ = 3.6 s, τ_2_ = 21.0 s. *AAV-Opn4L*: τ_1_ = 2.7 s, τ_2_ = 20.1 s. (**B**) Black traces in (**A**) superimposed for comparison. (**C**) Collected data on τ_1_ and τ_2_ and time-to-peak from individual cells of each genotype. Bars and error bars represent mean ± SD. (n.s., not statistically significant).

We have examined the waveform of the dim-flash responses more closely. In the linear range, these waveforms provide basic information about phototransduction^13,22^. Previously, we have shown that these responses from M1-ipRGCs can be described by the convolution of two single-exponential decays^10^. We confirmed the same here. Furthermore, we did not observe any obvious difference between the WT response and those from OPN4S- or OPN4L-expressing M1-ipRGCs. Figure 2A, top to bottom, shows the normalized, grand-average dim-flash response (black) from nine WT (with individual cell’s average response shown in grey), ten OPN4S-, and nine OPN4L-expressing M1-ipRGCs (room temperature) elicited by a small ~ 40-µm light spot. They are magnified and overlaid in Fig. 2B, showing their strong similarity. Before normalization, the responses to individual flash trials were 5–15 pA, well within the linear range. The red traces in Fig. 2A are respective fits by the convolution of two single-exponential declines, $e^{{ - t/\tau_{1} }} {*}e^{{ - t/\tau_{2} }}$, equal to $A\left( {e^{{ - t/\tau_{1} }} - e^{{ - t/\tau_{2} }} } \right)$, where A is a proportionality constant, with τ_1_ = 2.7 s, τ_2_ = 22.9 s for WT cells, τ_1_ = 3.6 s, τ_2_ = 21.0 s for OPN4S-expressing cells, and τ_1_ = 2.7 s, τ_2_ = 20.1 s for OPN4L-expressing cells (room temperature). In Fig. 2C, the comparisons between the three genotypes are provided more quantitatively by the mean ± SD of τ_1_ and τ_2_ values from fits to individual cells (WT: τ_1_ = 3.1 ± 1.5 s and τ_2_ = 21.5 ± 4.6 s (n = 9), OPN4S: τ_1_ = 3.9 ± 1.2 s and τ_2_ = 20.6 ± 4.3 s (n = 10), and OPN4L: τ_1_ = 3.2 ± 1.5 s and τ_2_ = 18.5 ± 2.9 s (n = 9)), with no significant difference across the three cell groups (*p* = 0.39 for τ_1_ and *p* = 0.23 for τ_2_, Kruskal–Wallis test). There is also no significant difference in the time-to-peak of the dim-flash responses (Fig. 2C, 6.6 ± 1.9 s, 7.4 ± 1.5 s and 6.2 ± 1.9 s, respectively; *p* = 0.34, Kruskal–Wallis test).

In summary, neither dim-flash sensitivity nor dim-flash response kinetics triggered by OPN4S versus OPN4L appeared to be very different from each other.

### Single-photon response

**Figure 3:**
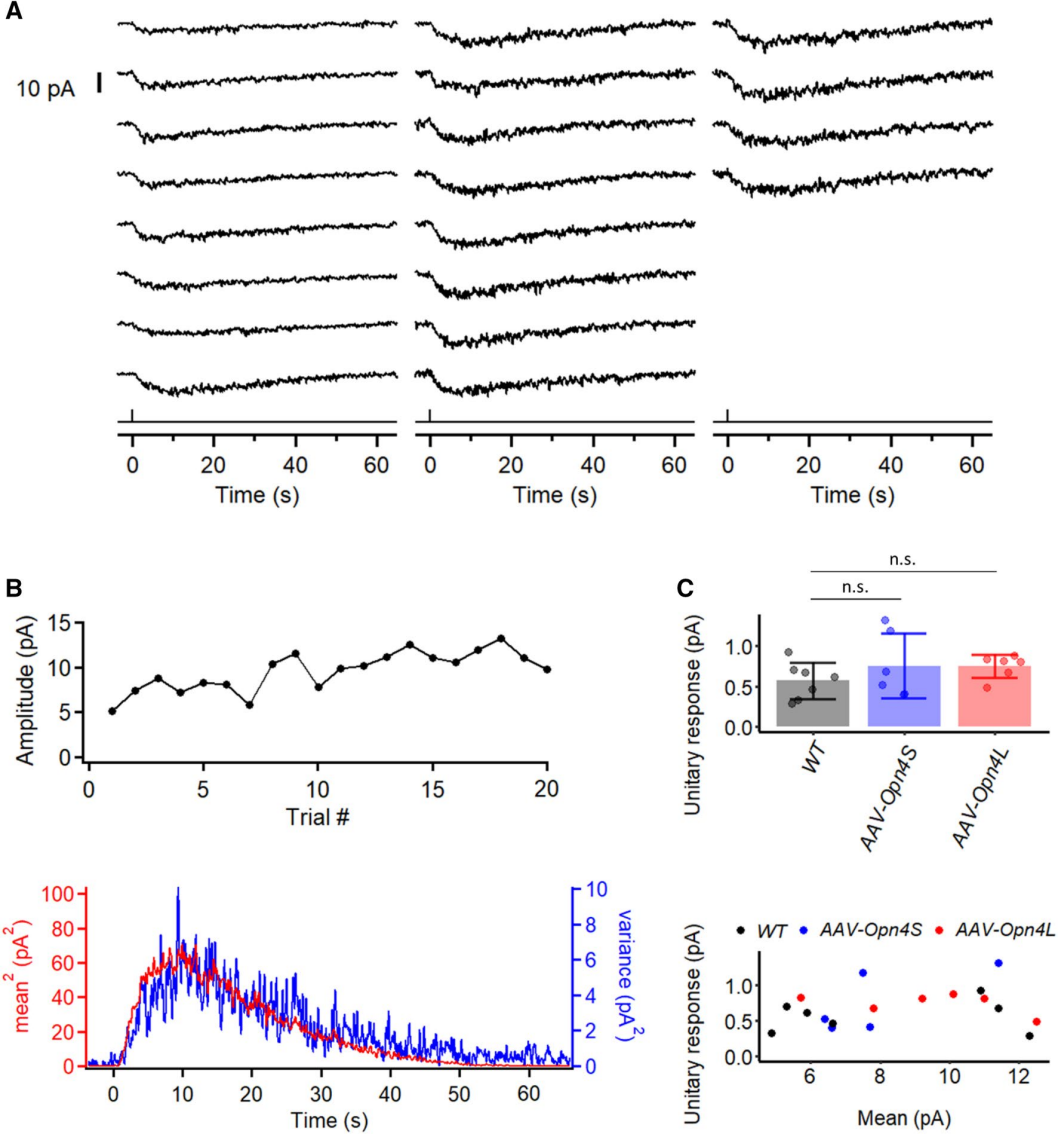
Estimation by fluctuation analysis of intrinsic single-photon response in M1-ipRGCs expressing different melanopsin isoforms. (**A**) A series of responses to identical dim flashes recorded from an Opn4L-expressing (*AAV-Opn4L*) M1-ipRGC. 50-ms, 480-nm flashes at 9.8 × 10^4^ photons μm^−2^ was delivered in a 40-μm spot centered at the soma. (**B**) Extraction of unitary response amplitude from an *AAV-Opn4L* M1-ipRGC. **Top**: Plot of successive response amplitudes over time to indicate stationarity. **Bottom**: Similar waveforms shown by the square of the ensemble mean of a series of responses to identical dim flashes and the ensemble variance of the dim-flash responses. (**C**) **Top**: Comparison of the unitary-response amplitude from WT M1-ipRGCs (n = 7), *AAV-Opn4S* M1-ipRGCs (n = 6), and *AAV-Opn4L* M1-ipRGCs (n = 6). (n.s. not statistically significant) **Bottom**: unitary response amplitude from individual cells plotted against their ensemble mean.

Previously, we have shown that WT mouse M1-ipRGCs respond to a single absorbed photon with a signal large enough to be resolved^10^. To see whether this unitary-response amplitude is any different originating from OPN4S versus OPN4L, we used noise analysis to address this question. We illuminated an M1-ipRGC with repeated identical dim flashes (via a light spot of ~ 40-μm diameter centered on soma) to elicit an ensemble of responses (typically 20–50 trials) in the linear amplitude range at room temperature, with an example from OPN4L-expressing M1-ipRGCs shown in Fig. 3A. Apart from random, trial-to-trial fluctuations in the responses, the recordings were fairly stable over time (Fig. 3B, top). Typically, over time, there was a slight, progressive increase in the response amplitude as well as an emergence of some higher-frequency baseline noise. The progressive increase in response amplitude could be due to recovery from some light adaptation caused by the initial light required for identifying the cell based on tdTomato and GFP-fluorescence. The reason for the emergence of the higher-frequency baseline noise is unclear. The response ensemble mean, *m*(*t*), and variance, *σ*^2^(*t*), were computed from the collected responses. The time courses of [*m*(*t*)]^2^ and *σ*^2^(*t*) overlapped fairly well, consistent with the number of unitary responses occurring per flash trial varying randomly according to the Poisson distribution but the unitary responses themselves having a roughly constant waveform (Fig. 3B, bottom). As such, the *σ*^2^(*t*)/*m*(*t*) ratio at the transient peak of the response provides an estimate for the unitary-response amplitude, being 0.81 pA for the OPN4L-expressing cell shown in Fig. 3A. Collected *σ*^2^/*m* values are summarized in Fig. 3C, top, with mean ± SD = 0.57 ± 0.22 pA (n = 7) for WT M1-ipRGCs, 0.75 ± 0.40 pA (n = 6) for OPN4S-expressing cells, and 0.75 ± 0.14 pA (n = 6) for OPN4L-expressing cells. As expected, the value of *σ*^2^*/m* is independent of *m* within the linear range (Fig. 3C, bottom). The unitary response was seemingly larger for OPN4S- and OPN4L-expressing ipRGCs than WT ipRGCs, but this difference was not statistically significant (*p* = 0.33, Kruskal–Wallis test). Overall, our measurements were well within the range previously reported^10,12^.

In sum, the unitary-response waveform (see previous section) and amplitude both appeared to be similar between the OPN4S and OPN4L melanopsin isoforms, consistent with the dim-flash sensitivity results described earlier.

### Effect of mutating key C-terminal phosphorylation sites on melanopsin

In rod photoreceptors, the decay of the active form of rhodopsin (metarhodopsin II) takes minutes, followed by its release of the photoisomerized chromophore (all-*trans*-retinal) ‒ a process called photobleaching^23^. Long before this decay, however, the active rhodopsin is phosphorylated by rhodopsin kinase (also called G-protein-coupled-receptor kinase 1, or GRK1) at the C-terminus, which partially inactivates its signaling, followed by its complete inactivation upon the binding of rod arrestin (arrestin 1)^24,25^. As a result, rhodopsin molecules have an estimated average active lifetime of only 40–80 ms in mammalian rods^26,27^. The genetic deletion of rhodopsin’s C-terminal phosphorylation sites, or of rhodopsin kinase (GRK1), leads to rod dim-flash responses that are greatly prolonged^28,29^. In contrast, invertebrate visual pigments such as in *Drosophila* differ from rod and cone pigments in that they are bistable instead of being photobleachable^30^. Furthermore, although photoexcited *Drosophila* rhodopsin is also phosphorylated, to be followed by the binding of arrestin for inactivation, there is indication in this case that phosphorylation is not a prerequisite for arrestin binding^30^. Melanopsin is thought to be more homologous to invertebrate visual pigments than to rod and cone pigments. On the other hand, its photochemistry is quite complex by showing signs of tristability^31^. Regarding active-melanopsin inactivation, it does resemble the behavior of rod and cone pigments by going through the prerequisite phosphorylation followed by the binding of β-arrestins 1 and 2 (close homologs of rod and cone arrestins)^32^.

**Figure 4:**
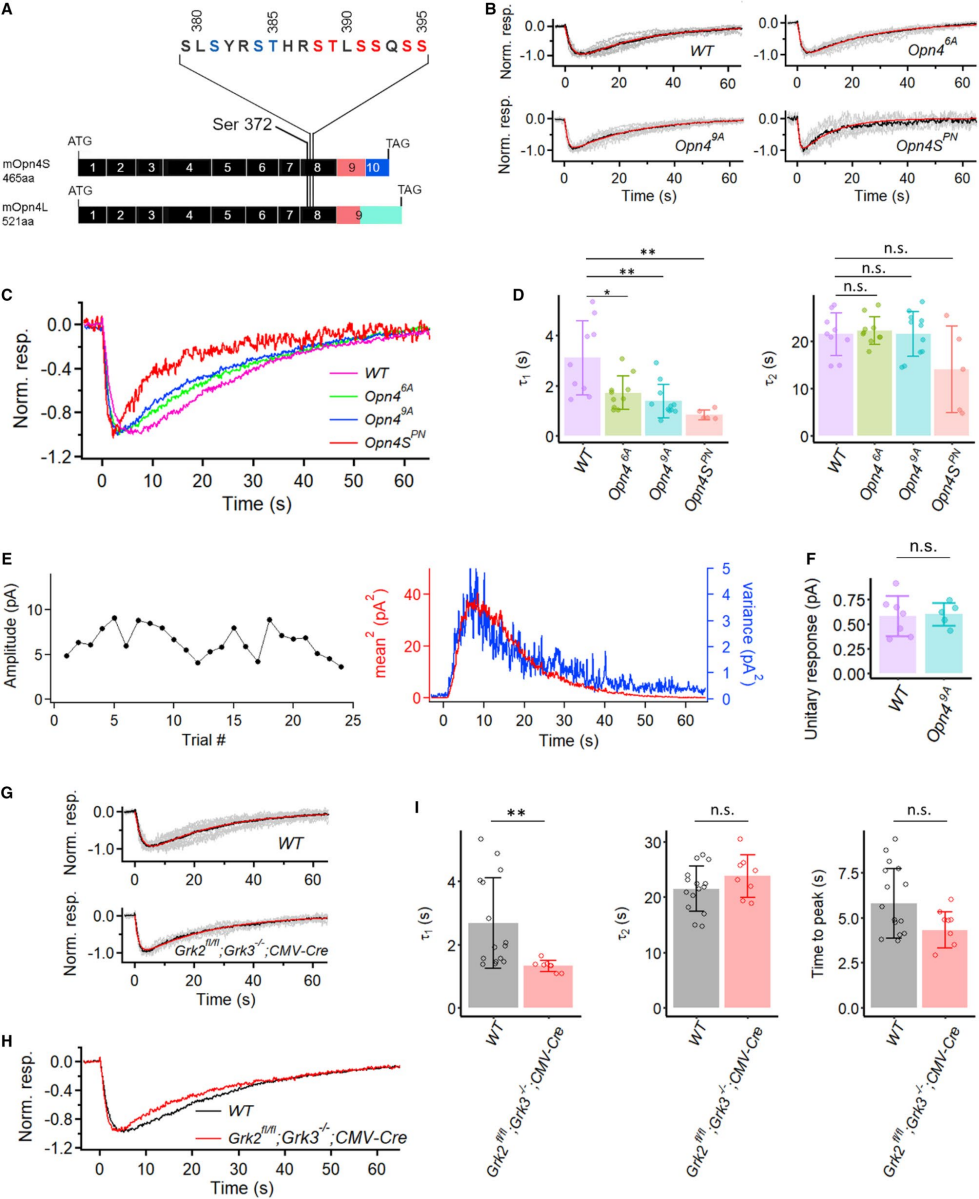
Intrinsic light responses from M1-ipRGCs expressing different phosphorylation-mutant melanopsins (*Opn4*^*6A*^, *Opn4*^*9A*^, *Opn4S*^*PN*^) and also from conditional GRK2, GRK3 double knock-out M1-ipRGCs. (**A**) Generation of *Opn4*^*6A*^ and *Opn4*^*9A*^ mice. The codon for Ser372, the most proximal potential phosphorylation site on the C-terminus of melanopsin, is located on exon 8. The six “critical phosphorylation sites” proposed by Blasic et al*.*^34^ are marked in red. Three additional residues proposed by Mure et al*.*^35^ are marked in blue. Note that these proposed “critical residues” are shared by both OPN4S and OPN4L. (**B**) Grand-averaged dim-flash response (black trace in each panel) from nine WT, ten *Opn4*^*6A*^, eleven *Opn4*^*9A*^, and five *Opn4S*^*PN*^ M1-ipRGCs. All cells were stimulated via a light spot of 40-μm diameter centered on soma. Superimposed grey traces in each panel show normalized and averaged responses from individual cells. The red trace in each panel shows the fit to the black trace by the convolution of two single-exponential decays, $e^{{ - t/\tau_{1} }} {*}e^{{ - t/\tau_{2} }}$, with τ_1_ = 2.7 s and τ_2_ = 22.9 s for WT, τ_1_ = 1.6 s and τ_2_ = 22.9 s for *Opn4*^*6A*^ cells, τ_1_ = 1.2 s and τ_2_ = 22.8 s for *Opn4*^*9A*^ cells, and τ_1_ = 0.81 s and τ_2_ = 11.5 s for *Opn4S*^*PN*^ cells. (**C**) Superposition of black traces in (**B**) for comparison of response waveforms in WT, *Opn4*^*6A*^, *Opn4*^*9A*^, and *Opn4S*^*PN*^ cells. (**D**) Collected data on τ_1_ and τ_2_ from individual cells of each genotype. Bars and error bars represent mean ± SD. WT: τ_1_ = 3.1 ± 1.5 s and τ_2_ = 21.5 ± 4.6 s. *Opn4*^*6A*^: τ_1_ = 1.7 ± 0.7 s and τ_2_ = 22.2 ± 2.9 s. *Opn4*^*9A*^: τ_1_ = 1.4 ± 0.7 s and τ_2_ = 21.5 ± 4.7 s. *Opn4S*^*PN*^: τ_1_ = 0.84 ± 0.20 s and τ_2_ = 14.0 ± 9.1 s. Adjusted *p*-values for pairwise comparisons in τ_1_ with the Benjamini–Hochberg procedure are 0.026 for WT vs. *Opn4*^*6A*^, 0.0053 for WT vs. *Opn4*^*9A*^, 0.0053 for WT vs. *Opn4S*^*PN*^. For comparisons in τ_2_: *p* = 0.28, Kruskal–Wallis test. (**, *p* < 0.01; *, *p* < 0.05; n.s., not statistically significant.) (**E**) Extraction of unitary response amplitude from an *Opn4*^*9A*^ M1-ipRGC. **Left:** Stationarity of the dim-flash response over time. **Right:** Similar waveforms shown by the square of the ensemble mean of a series of responses to identical dim flashes and the ensemble variance of the dim-flash responses. (**F**) Comparison between the unitary-response amplitudes from WT and *Opn4*^*9A*^ M1-ipRGCs. Bars and error bars represent mean ± SD. (**G**) Grand average (black trace) of normalized mean dim-flash responses (grey traces, 5–15 pA) from fifteen WT (top panel) and eight *CMV-Cre;Grk2*^*fl/fl*^*;Grk3*^*−/−*^ M1-ipRGCs (bottom panel). In each panel, the red trace is the fit by the convolution of two single-exponential declines, $e^{{ - t/\tau_{1} }} {*}e^{{ - t/\tau_{2} }}$. WT: τ_1_ = 2.2 s and τ_2_ = 23.3 s. *CMV-Cre;Grk2*^*fl/fl*^*;Grk3*^*−/−*^: τ_1_ = 1.3 s and τ_2_ = 23.8 s. (**H**) Superposition of grand-averaged dim-flash responses from WT and *CMV-Cre;Grk2*^*fl/fl*^*;Grk3*^*−/−*^ M1-ipRGCs (the two black traces in G) for comparison of the response waveforms. (**I**) Collected data for τ_1_, τ_2_ and time-to-peak from (G). τ_1_: 2.7 ± 1.4 s for WT and 1.3 ± 0.2 s for *CMV-Cre;Grk2*^*fl/fl*^*;Grk3*^*−/−*^ (*p* = 2.7 × 10^–4^, Wilcox test). τ_2_: 21.5 ± 4.1 s for WT and 23.8 ± 3.8 s for *CMV-Cre;Grk2*^*fl/fl*^*;Grk3*^*−/−*^ (*p* = 0.29, Wilcox test). Time-to-peak: 6.0 ± 1.9 s for WT and 4.6 ± 1.0 s for *CMV-Cre;Grk2*^*fl/fl*^*;Grk3*^*−/−*^ (*p* = 0.19, Wilcox test). (**, *p* < 0.01; n.s., not statistically significant).

Melanopsin possesses an unusually long C-terminal tail with many serine/threonine residues that can potentially be phosphorylated in situ. Blasic et al*.* first reported six serine/threonine residues on the C-terminus (Fig. 4A, marked in red) that are critical for melanopsin inactivation^33,34^. When heterologously expressed in HEK-293 cells and in response to light stimulation, a mutant melanopsin with these six residues replaced by alanine produced a more sustained elevation of intracellular Ca^2+^ (as an assay of melanopsin activity) than did WT melanopsin^34^. Upon mutating away all serine/threonine residues in the C-terminus, the same group found a similar phenotype^34^. Subsequently, another group^35^ used heterologous expression in CHO cells and also an assay of intracellular Ca^2+^ changes to indicate melanopsin activity, and found that nine serine/threonine residues within amino acid 380–397 (Fig. 4A, with residues marked in red and blue), including the six residues mentioned earlier and covering a highly-conserved region across species, are critical for melanopsin inactivation.

To check whether phosphorylation at these sites plays a role in determining the kinetics of the dim-flash response in M1-ipRGCs, we first mutated the six serine/threonine residues^34^ to alanine in mice with CRISPR (Fig. 4A; “Methods” section; mutant denoted as *Opn4*^*6A*^, short for homozygous *Opn4*^*6A/6A*^). This mutation is shared by OPN4S and OPN4L, thus the mutation was simultaneously created in both isoforms. Surprisingly, the dim-flash responses from homozygous *Opn4*^*6A*^ M1-ipRGCs were largely similar to those recorded from WT M1-ipRGCs (Fig. 4B, 4C), with no sign of an expected prolongation. The average τ_1_ from the *Opn4*^*6A*^ M1-ipRGCs (1.7 ± 0.7 s, n = 10) is smaller than that from WT cells (3.1 ± 1.5 s, n = 9; Fig. 4D), but the τ_2_ value is very similar across genotypes. We next mutated with CRISPR all nine serine/threonine residues within the conserved region, also common to OPN4S and OPN4L^35^ (Fig. 4A; “Methods” section; mutant denoted as *Opn4*^*9A*^*,* short for homozygous *Opn4*^*9A/9A*^). The τ_1_ from the *Opn4*^*9A*^ M1-ipRGCs (1.4 ± 0.7 s, n = 10) gets even smaller. τ_2_, however, remains largely the same as WT (see figure legend). For the phospho-null mutant, we aimed at achieving a mouse genotype with all C-terminal serines/threonines mutated to alanine, but did not succeed. Instead, we used AAV to express in *Opn4*^*−/−*^ M1-ipRGCs a phospho-null OPN4S protein (*AAV2-hSyn-Opn4S*^*PN*^*-IRES-GFP-WPRE*; “Methods” section), which has all twenty-nine serines/threonines in the short-isoform C-terminus mutated away. The infected *Opn4*^*−/−*^ M1-ipRGCs expressing OPN4S^PN^ protein gave dim-flash responses again similar to those of *Opn4*^*9A*^ M1-ipRGCs. τ_2_ appears shorter, although again with no statistical difference from WT (Fig. 4D). As long as light stimulation was restricted to dim flashes (otherwise see below), the *Opn4*^*9A*^ M1-ipRGCs behaved quite stably, to the extent that it was possible to elicit as many as over 20 dim-flash responses (Fig. 4E), with noise analysis giving a unitary response of 0.62 pA, not very different from WT M1-ipRGCs. Collected data gave 0.60 ± 0.11 pA (n = 5) (Fig. 4F).

Prior work by others^33,35,36^ suggests that the removal of phosphorylation of active melanopsin should prolong its light-triggered activity, to be reflected by a more prolonged time course of the dim-flash response. The simplest explanation for our not observing this behavior would be that the melanopsin phosphorylation step was perhaps quite fast even after its prolongation by phosphorylation removal, thus was masked by the dominant slow steps (τ_1_ and τ_2_) in the response kinetics. We also noticed that, in contrast to WT or *Opn4*^*6A*^ M1-ipRGCs, the *Opn4*^*9A*^ M1-ipRGCs and *Opn4S*^*PN*^ M1-ipRGCs showed a considerable delay in recovering their sensitivity after a prior bright flash, as if active melanopsin or its downstream phototransduction signaling required the pigment’s C-terminal phosphorylation in order to return to its initial resting state in darkness. Indeed, after a bright flash (10^7^–10^8^ photons μm^−2^, which was brighter than one producing a linear response), an *Opn4S*^*PN*^-M1 ipRGC would take as long as 1 h before being capable of responding to a dim flash again (versus ~ 5 min for a similarly-treated WT M1-ipRGC).

We checked whether deleting the relevant kinases had the same effect as mutating away the serines/threonines. Melanopsin has been demonstrated by co-immunoprecipitation to interact preferentially with GRK2 and GRK3, both of which are found in ipRGCs^33^. We introduced Cre-GFP into *Grk2*^*fl/fl*^*;Grk3*^*−/−*^ retina via adeno-associated virus serotype 2 (*AAV2-CMV-Cre-GFP*; UNC Vector Core). Compared to WT cells, successfully-infected M1-ipRGCs as revealed by GFP fluorescence showed dim-flash responses with unchanged τ_2_ but a significantly shortened τ_1_, rather similar to the *Opn4*^*9A*^ and *Opn4S*^*PN*^ phenotypes (Fig. 4G–I).

**Figure 5:**
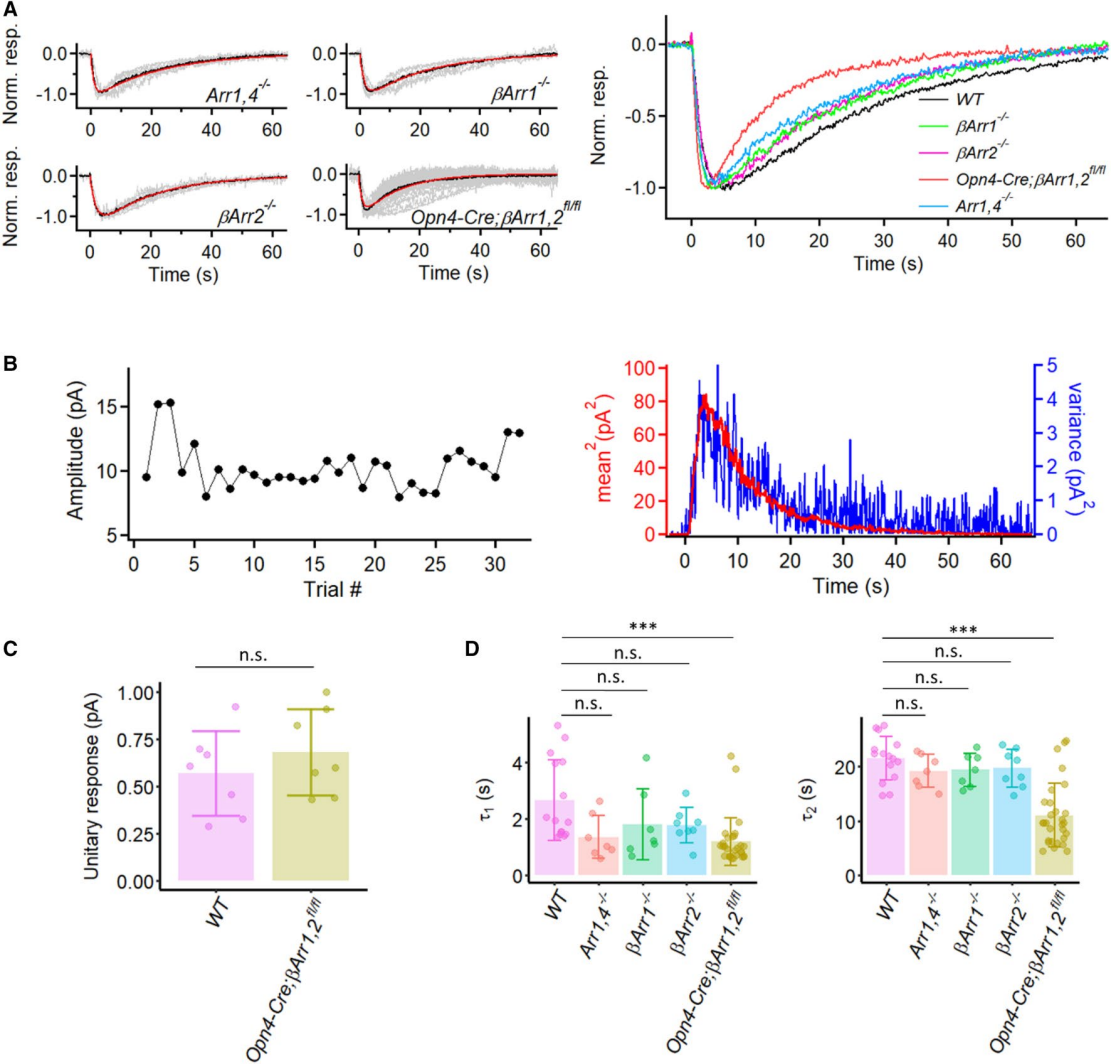
Kinetics of intrinsic dim-flash responses from various arrestin knock-out M1-ipRGCs. (**A**) **Left:** Grand average (black trace) of normalized mean dim-flash responses (grey traces, 5–15 pA) from seven *Arr1,4*^*−/−*^ M1-ipRGCs, seven *βArr1*^*−/−*^ M1-ipRGCs, eight *βArr2*^*−/−*^ M1-ipRGCs, and twenty-nine *Opn4-Cre;βArr1,2*^*fl/fl*^ M1-ipRGCs. In each panel, the red trace is the fit to the black trace by the convolution of two single-exponential declines, $e^{{ - t/\tau_{1} }} {*}e^{{ - t/\tau_{2} }}$. *Arr1,4*^*−/−*^: τ_1_ = 1.0 s, τ_2_ = 22.3 s. *βArr1*^*−/−*^: τ_1_ = 1.5 s, τ_2_ = 21.1 s. *βArr2*^*−/−*^: τ_1_ = 2.0 s, τ_2_ = 18.9 s. *Opn4-Cre;βArr1,2*^*fl/fl*^: τ_1_ = 1.1 s, τ_2_ = 12.5 s. **Right:** Superposition of grand-averaged dim-flash responses from *Arr1,4*^*−/−*^, *βArr1*^*−/−*^, *βArr2*^*−/−*^, *Opn4-Cre;βArr1,2*^*fl/fl*^ M1-ipRGCs (the black traces in **Left** panel), as well as WT M1-ipRGCs for comparison of the response waveforms. (**B**) Extraction of unitary response amplitude from an *Opn4-Cre;βArr1,2*^*fl/fl*^ M1-ipRGC. **Left:** Stationarity of the dim-flash response over time. **Right:** Similar waveforms shown by the square of the ensemble mean of a series of responses to identical dim flashes and the ensemble variance of the dim-flash responses. Unitary response = 0.43 pA. (**C**) Comparison between the unitary-response amplitudes from WT and *Opn4-Cre;βArr1,2*^*fl/fl*^ M1-ipRGCs. Bars and error bars represent mean ± SD. WT: 0.57 ± 0.22 pA (n = 7). *Opn4-Cre;βArr1,2*^*fl/fl*^: 0.68 ± 0.23 pA (n = 7). *p* = 0.62, Wilcoxon test. (n.s., not statistically significant.) (**D**) Collected data on τ_1_ and τ_2_ from individual cells of each genotype. Bars and error bars represent mean ± SD. WT: τ_1_ = 2.7 ± 1.4 s and τ_2_ = 21.5 ± 4.1 s. *Arr1,4*^*−/−*^: τ_1_ = 1.4 ± 0.8 and τ_2_ = 19.2 ± 3.0 s. *βArr1*^*−/−*^: τ_1_ = 1.8 ± 1.3 s and τ_2_ = 19.4 ± 3.1 s. *βArr2*^*−/−*^: τ_1_ = 1.8 ± 0.6 s and τ_2_ = 19.7 ± 3.4 s. *Opn4-Cre;βArr1,2*^*fl/fl*^: τ_1_ = 1.2 ± 0.8 s and τ_2_ = 11.0 ± 5.9 s. Adjusted *p*-values for pairwise comparisons in τ_1_ using Benjamini–Hochberg procedure are 0.06 for WT vs. *Arr1,4*^*−/−*^, 0.18 for WT vs. *βArr1*^*−/−*^, 0.56 for WT vs. *βArr2*^*−/−*^, 1.0 × 10^–4^ for WT vs. *Opn4-Cre;βArr1,2*^*fl/fl*^. Adjusted *p*-values for pairwise comparisons in τ_2_ using Benjamini–Hochberg procedure are 0.44 for WT vs. *Arr1,4*^*−/−*^, 0.44 for WT vs. *βArr1*^*−/−*^, 0.46 for WT vs. *βArr2*^*−/−*^, 1.6 × 10^–4^ for WT vs. *Opn4-Cre;βArr1,2*^*fl/fl*^. (***, *p* < 0.001; n.s., not statistically significant).

We have also examined the effect of genetically removing the rod arrestin (Arrestin 1) and cone arrestin (Arrestin 4) on M1-ipRGCs’ dim-flash response, but did not observe any significant effect (Fig. 5A, right panel and 5D). Although τ_1_ seems to be smaller than that of WT on the plot, these experiments were mostly done by using a different light source, especially for initially identifying the cells. WT data from the second light source also showed a slightly shortened τ_1_, possibly caused by different light history (data not shown). Cameron and Robinson (2014) have reported the presence of β-arrestins 1 and 2 (Arrestins 2 and 3) in ipRGCs. Accordingly, we examined their knockout effect as well. At least with dim flashes, the *βArr1*^*−/−*^ and *βArr2*^*−/−*^ single-knockout response waveforms were roughly normal (Fig. 5A,D). However, the *Opn4-Cre;βArr1,2*^*fl/fl*^ double-knockout response waveform showed a somewhat more dramatic behavior reminiscent of the *Opn4S*^*PN*^ phenotype (see Fig. 4), with shorter *τ*_*1*_ and *τ*_*2*_, as well as a shorter response time-to-peak than normal (Fig. 5A,D). On the other hand, the unitary-response amplitude derived from dim flashes seemed normal (Fig. 5B,C).

At this point, it is difficult to reconcile our receptor-current findings on the deletion effects of melanopsin phosphorylation or β-arrestins 1 and 2 with experiments by others using cultured cells^32^ or action-potential recordings^37^. Given the additional complexity of melanopsin’s unusual photochemistry with three stable states^31^, we have decided not to pursue this question further here.

### G-protein deactivation: GAP activity of PLCβ4

In trimeric G-protein signaling, the hydrolysis of the GTP bound to the activated G-protein α-subunit (Gα*-GTP) to GDP, thereby deactivating Gα (Gα-GDP), typically involves a GTPase-activating protein (GAP) complex. In rod phototransduction, this GAP complex comprises the cGMP-phosphodiesterase, which is the effector enzyme downstream of the rod G protein (transducin), together with RGS9 (Regulator of G protein Signaling-9), R9AP (RGS9-anchor protein), and Gβ_5_, an orphan G protein β-subunit not found in heterotrimeric G proteins. Disruption of the GAP complex slows down G-protein deactivation, as found in *Rgs9*^*−/−*^ rods, leading to a prolonged flash response^38^.

**Figure 6:**
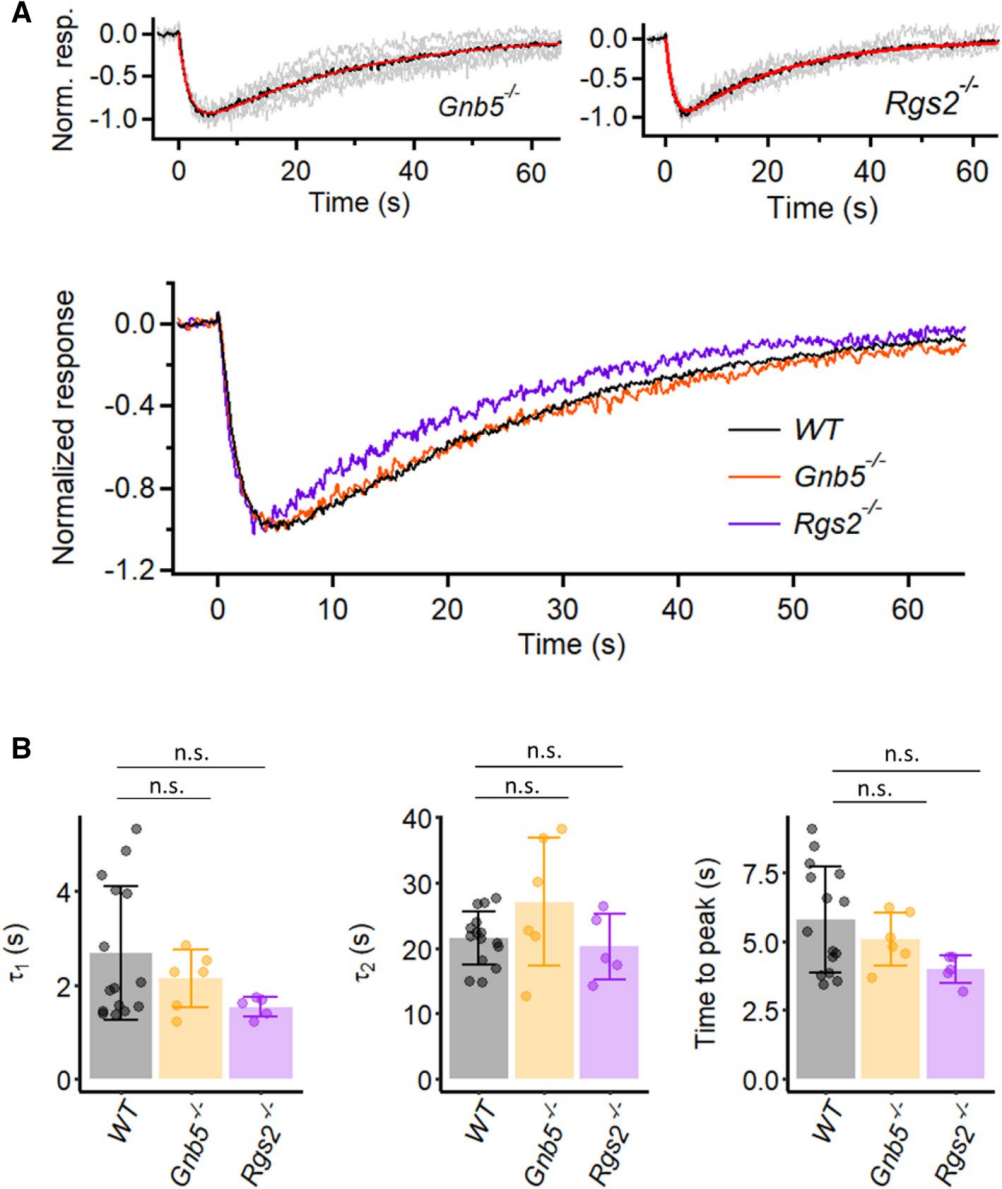
Kinetics of intrinsic dim-flash responses from *Gnb5*^*−/−*^ M1-ipRGCs and *Rgs2*^*−/−*^ M1-ipRGCs. (**A**) **Top:** Grand average (black trace) of normalized mean dim-flash responses (grey traces, 3–15 pA) from six *Gnb5*^*−/−*^ M1-ipRGCs and four *Rgs2*^*−/−*^ M1-ipRGCs. In each panel, the red trace is the fit to the black trace by the convolution of two single-exponential declines, $e^{{ - t/\tau_{1} }} {*}e^{{ - t/\tau_{2} }}$. *Gnb5*^*−/−*^: τ_1_ = 1.87 s, τ_2_ = 26.8 s. *Rgs2*^*−/−*^: τ_1_ = 1.51 s, τ_2_ = 20.0 s. **Bottom:** Superposition of grand-averaged dim-flash responses from WT (same trace in Fig. 2B), *Gnb5*^*−/−*^ and *Rgs2*^*−/−*^ M1-ipRGCs (the black traces in **Top**) for comparison of the response waveforms. (**B**) Collected data on τ_1_ and τ_2_ and time-to-peak from individual cells of each genotype. Bars and error bars represent mean ± SD. τ_1_: 2.1 ± 0.6 s for *Gnb5*^*−/−*^, 1.5 ± 0.2 s for *Rgs2*^*−/−*^, and 2.7 ± 1.4 s for WT (*p* = 0.25, Kruskal–Wallis test). τ_2_: 27.1 ± 9.9 s for *Gnb5*^*−/−*^, 20.2 ± 5.0 s for *Rgs2*^*−/−*^, and 21.5 ± 4.1 s for WT (*p* = 0.34, Kruskal–Wallis test). Time to peak: 5.3 ± 1.0 s for *Gnb5*^*−/−*^, 4.2 ± 0.5 s for *Rgs2*^*−/−*^, and 6.0 ± 1.9 s for WT (*p* = 0.09, Kruskal–Wallis test). (n.s., not statistically significant).

RGS9 belongs to a large RGS protein family with many members acting as GAPs for accelerating the termination of GPCR signaling^39,40^. We attempted to check the existence of a GAP in M1-ipRGCs. The R7 family, an RGS subfamily comprising RGS6, RGS7, RGS9 and RGS11, is a testable candidate because RGS7 and RGS11 in conjunction with Gβ_5_ also serve as a GAP in the signaling from photoreceptor to retinal ON-bipolar cells via a Gα_o_-mediated metabotropic-glutamatergic (mGluR6) synapse^41,42^. Genetically deleting Gβ_5_ (*Gnb5*^*−/−*^) eliminates expression of all R7-subfamily RGS proteins^43^. We followed this strategy with *Gnb5*^*−/−*^ M1-ipRGCs, but found the dim-flash response to be normal (Fig. 6A,B). We next checked RGS2, which is reported to be specific for the Gα_q_-subfamily of G proteins (Kimple et al., 2011), thus appropriate for regulating M1-ipRGC phototransduction^15^. However, the dim-flash response of *Rgs2*^*−/−*^ M1-ipRGCs did not show any prolongation either (Fig. 6A,B).

Over twenty RGS family members are known to exist, with many acting potentially on the Gα_q_-subfamily^40^. In view of this complexity, we decided instead to take the alternative approach of focusing on the effector enzyme, PLCβ4, downstream of Gα_q,11,14_ in M1-ipRGC phototransduction^11^. In *Drosophila* phototransduction, the PLC effector enzyme downstream of Gα_q_ acts as a GAP^44^, and so do its multiple mammalian homologs, PLCβ1-4^39,45^. Moreover, a highly-conserved asparagine residue in *Drosophila* PLC and mammalian PLCβ isoforms has been found to be important for this GAP activity, such that mutating it to alanine specifically abolishes the enzyme’s GAP activity without affecting its activation by Gα_q_ or its catalytic activity^46^.

**Figure 7:**
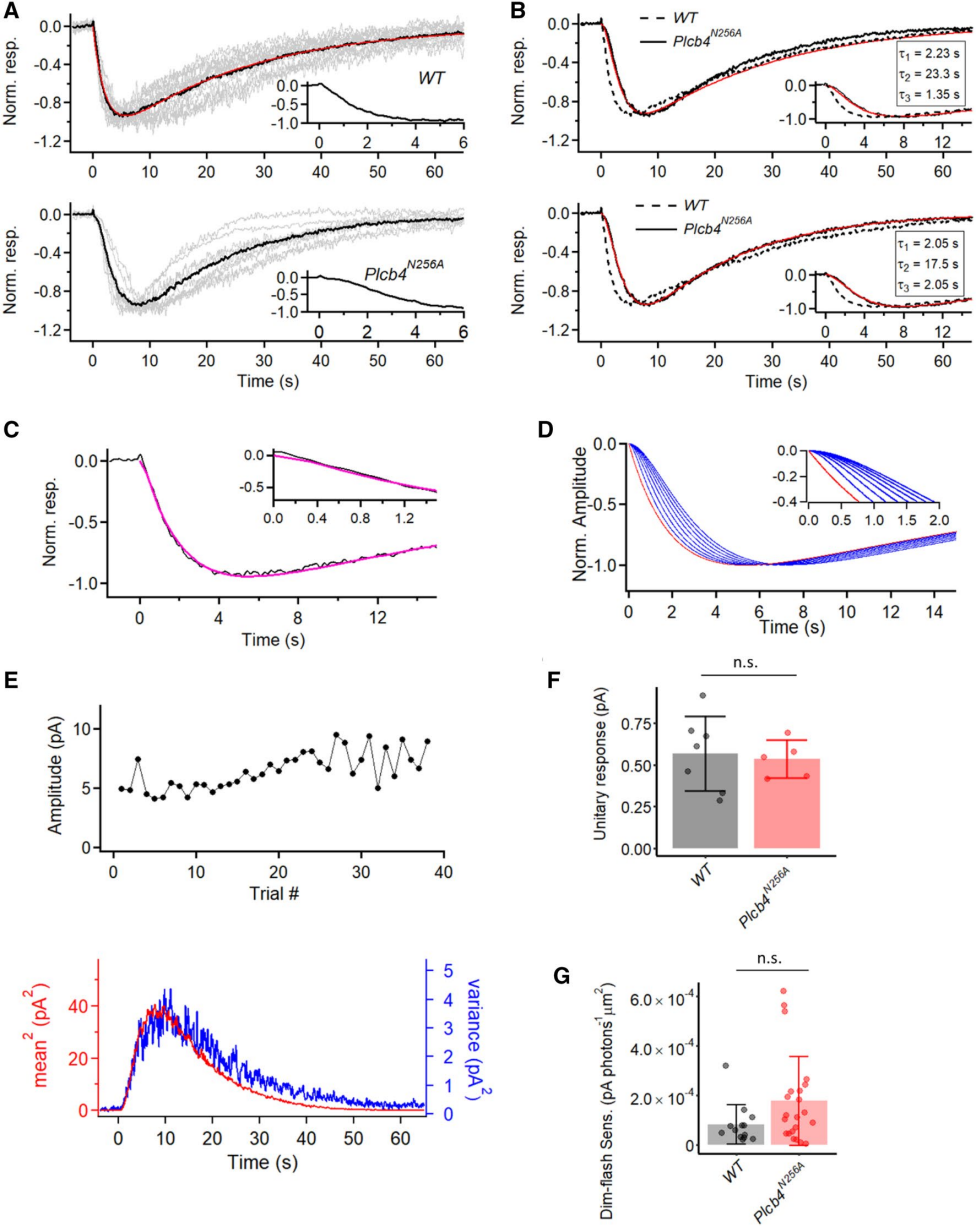
Characterization of intrinsic dim-flash responses in *Plcb4*^*N256A*^ M1-ipRGCs. (**A**) Grand average (black trace) of normalized mean dim-flash responses (grey traces) from fifteen WT M1-ipRGCs (top) and nine *Plcb4*^*N256A*^ M1-ipRGCs (bottom). Insets show the first 6 s of the light response for better visualization of onset. In top panel, the red trace is the fit to the black trace by the convolution of two single-exponential declines, $e^{{ - t/\tau_{1} }} {*}e^{{ - t/\tau_{2} }}$. with τ_1_ = 2.23 s, τ_2_ = 23.3 s (red). (**B**) Fitting traces (red) of the grand average trace (black) from nine *Plcb4*^*N256A*^ M1-ipRGCs to the convolution of three exponential functions, $e^{{ - t/\tau_{1} }} {*}e^{{ - t/\tau_{2} }} *e^{{ - t/\tau_{3} }}$. Top: τ_1_ and τ_2_ are fixed to 2.23 s and 23.3 s, while we look for the τ_3_ value that gives the best fit. Bottom: another fitting result with no constraints placed on τ_1_, τ_2_ and τ_3_. With these two fitting methods, we estimate the additional time constant in the *Plcb4*^*N256A*^ response waveform (denoted τ_3_) to be on the order of magnitude of 1.35–2.05 s. The grand average response waveform from WT cells is shown in dashed line for comparison. (**C**) Fitting trace (megenta) of grand average (black) of normalized dim-flash responses from WT M1-ipRGCs to the convolution of three exponential functions, $e^{{ - t/\tau_{1} }} {*}e^{{ - t/\tau_{2} }} *e^{{ - t/\tau_{3} }}$, with τ_1_ = 1.96 s; τ_2_ = 23.6 s; τ_3_ = 0.18 s. Inset shows the first 1.5 s of the response for better visualization of the onset. (**D**) Normalized function curves of the convolution of three exponential functions, $e^{{ - t/\tau_{1} }} {*}e^{{ - t/\tau_{2} }} *e^{{ - t/\tau_{3} }}$. τ_1_ and τ_2_ are fixed to 1.96 s and 23.6 s, according to the fitting results in (**C**). The sigmoidal shape of the onset (shown in inset) changes accordingly with the increase of τ_3_. Red trace: when τ_3_ = 0 s; Blue traces from left to right in sequence: when τ_3_ = 0.2, 0.4, 0.6, 0.8, 1.0, 1.2, and 1.4 s. (**E**) Extraction of unitary response amplitude from a *Plcb4*^*N256A*^ M1-ipRGC. **Top:** Stationarity of the dim-flash response over time. **Bottom:** Similar waveforms shown by the square of the ensemble mean of a series of responses to identical dim flashes and the ensemble variance of the dim-flash responses. (**F**) Comparison between the unitary-response amplitudes from WT and *Plcb4*^*N256A*^ cells. Bars and error bars represent mean ± SD. WT: 0.57 ± 0.22 pA (n = 7). *Plcb4*^*N256A*^: 0.53 ± 0.11 pA (n = 5). *p* = 0.76, Wilcoxon test. (n.s., not statistically significant.) (**G**) Comparison between the dim-flash sensitivity from WT and *Plcb4*^*N256A*^. Bars and error bars represent mean ± SD. WT: 8.2 ± 8.0 × 10^–5^ pA photons^-1^ µm^2^ (n = 13). *Plcb4*^*N256A*^: 1.76 ± 1.81 × 10^–4^ pA photons^-1^ µm^2^ for (n = 22); *p* = 0.14, Wilcoxon test. (n.s., not statistically significant).

We introduced the above GAP-deficient point mutation into mouse PLCβ4 (N256A) by CRISPR (*Plcb4*^*N256A*^, short for homozygous *Plcb4*^*N256A/N256A*^; “Methods” section). Indeed, although the dim-flash response of *Plcb4*^*N256A*^ M1-ipRGCs showed no difference from WT in its decline phase (i.e., not prolonged), it invariably showed a mild sigmoidal shape at its onset (Fig. 7A, bottom inset) ‒ a feature absent in WT (Fig. 7A, top inset), resulting in a slightly longer time-to-peak and also a slightly longer 10–90% rise time in the *Plcb4*^*N256A*^ response (Fig. S2A and B). As such, whereas the waveform of the WT dim-flash response is described by the convolution of two single-exponential decays (Fig. 7A, top), an additional single-exponential time constant, τ_3_, has to be introduced for describing the mutant response’s sigmoidal rise (Fig. 7B). We evaluated τ_3_ in two ways. The first (Fig. 7B, top) was to adopt the average τ_1_ and τ_2_ values previously derived from fitting the WT dim-flash-response waveform (τ_1_ = 2.23 s, τ_2_ = 23.3 s; see Fig. 2C and associated text), then to ask what value τ_3_ has to take on in order to provide a reasonable fit to the mutant response waveform. As such, we obtained τ_3_ = 1.35 s. The second way (Fig. 7B, bottom) was to let τ_1_, τ_2_ and τ_3_ be free parameters for fitting the mutant response waveform, from which we obtained τ_1_ = 2.05 s, τ_2_ = 17.5 s and τ_3_ = 2.05 s. The two τ_3_ values gave a mean of 1.7 s. We interpret this time constant to represent the abnormally slow decay of activated Gα_q_*-GTP to Gα_q_-GDP when GAP activity is defective. It is worth pointing out here that, in the mathematical fits described in this work, the parameters τ_1_, τ_2_ and τ_3_ are commutative. In other words, the resulting waveform is the same regardless of their physical order of occurrence in the pathway. Because the values of τ_1_ and τ_3_ are quite similar, it is more appropriate to view them as being broadly comparable than to take on further distinction between them. In M1-ipRGCs, the three Gα_q_-subfamily members, namely, Gα_q_, Gα_11_, and Gα_14_, all participate in phototransduction^15^ but may not necessarily all deactivate at exactly the same rate. Thus, it is probably a weighted mean that we were measuring. The question remains: How fast is the deactivation of Gα_q_, Gα_11_, and Gα_14_ under normal GAP activity? To address this question, we tried to fit the dim-flash response waveform of WT M1-ipRGCs (Fig. 7A) with three, instead of two, time constants as free parameters, we obtained τ_1_ = 1.96 s, τ_2_ = 23.6 s and τ_3_ = 0.18 s at room temperature (Fig. 7C). Taking this approach and treating the shortest time constant from this fit to be τ_3_ (note that this distinction may not be appropriate for *Plcb4*^*N256A*^ cells, where the shortest and second shortest time constants are often fairly close)_,_ then τ_3_ turned out to be 0.11 ± 0.056 s in WT cells (mean ± SD, n = 15), in stark contrast to 1.9 ± 0.91 s in *Plcb4*^*N256A*^ cells (mean ± SD, n = 9; Fig. S2C). In a sense, this non-zero τ_3_ is a hypothetical value that, at least in principle, is capable of retaining a reasonable fit to the recorded WT response, although its true value is not really known. Fixing τ_1_ and τ_2_ at their average WT values (Fig. 7B,C legend) and letting τ_3_ vary from 0 ‒ 1.4 s in 0.2-s increments, we found that the sigmoidicity became progressively overly prominent very quickly (Fig. 7D). Thus, although τ_3_ can be between 0 and 0.2 s, it cannot be any larger. Of course, strictly speaking, τ_3_ may not necessarily correspond to the WT GAP time constant, but can be any fast step in the phototransduction pathway.

The unitary-response amplitude of *Plcb4*^*N256A*^ M1-ipRGCs extracted from noise analysis is largely similar to that of WT M1-cells, with an amplitude of 0.53 ± 0.11 pA (mean ± SD, n = 5) (Fig. 7E,F). The slight disagreement in shape between the square of the ensemble mean response and the ensemble variance of the response possibly implicates a slight statistical variability in the speed of termination of the unitary response due to the absence of GAP activity. The dim-flash sensitivity also remains statistically unchanged when comparing with that of WT M1-cells (see figure legend) (Fig. 7G).

## Discussion

In this work, we did not find any obvious difference in waveform between the dim-flash responses originating from the OPN4S and OPN4L melanopsin isoforms in M1-ipRGCs. Both are literally indistinguishable from WT. The unitary-response amplitude was likewise similar between them. As pointed out in “Results” section, we are focusing here on basic phototransduction aspects of ipRGCs as revealed by their dim-flash responses rather than signaling properties under specific conditions such as prolonged illumination. At the same time, the differential changes in non-image-forming visual behaviors observed after disruption of one melanopsin isoform or another as reported by Jagannath et al*.*^18^ may not arise necessarily from mechanisms associated with specific phototransduction characteristics of OPN4S or OPN4L. Instead, the differences could come from firing properties of ipRGCs, either intrinsic to particular ipRGC subtypes or driven by the responses from Opn4S versus Opn4L, in light situations that we have not examined^47^. Finally, there could be pre- or postsynaptic mechanisms at the ipRGC axonal terminals on a given brain target^48^.

The lack of intrinsic-response prolongation that we observed after deletion of the critical C-terminal phosphorylation sites on melanopsin, or of β-arrestin 1 and 2, is not expected given the previous findings by others^34,35^. One possibility is that melanopsin in heterologous expression in culture cells behaves differently from that in ipRGCs. Another possibility is that the effect of melanopsin phosphorylation and the subsequent binding of β-arrestin 1 and 2 (Ref. 32,37) are below our temporal resolution if their associated kinetics are much faster than the dominant time constants (τ_1_ and τ_2_) for the response waveform. In any case, the complexity of the termination of melanopsin activity is presently unresolvable based on the waveform of the physiological dim-flash response.

Perhaps the most interesting finding of this work is a revelation of the deactivation of Gα_q_, Gα_11_ and Gα_14_ in M1-ipRGC phototransduction involving GAP activity. We have demonstrated that PLCβ4, the effector enzyme in M1-ipRGC phototransduction, indeed participates in GAP activity, and we verified that its amino-acid residue 256, an asparagine, is crucial for this activity. With the gene-knockout strategy, we ruled out the involvement of Gβ_5_ (the only Gβ-subunit known not to be a constituent of heterotrimeric G proteins), along with RGS6, 7, 9 and 11 (see “Results” section), for GAP activity in M1-ipRGC phototransduction. Separately, we found RGS2, a GAP protein thought to be for Gα_q_-subfamily members, also dispensable. The study of GAP mechanisms is an active field, with much remaining unknown regarding the composition of particular GAP complexes that terminate G-protein activity in different pathways, except for the well-known case of rod/cone phototransduction, which involves four proteins in a complex as described earlier for transducin.

From waveform fitting, the WT dim-flash response may in principle contain another time constant as long as it is no larger than perhaps 0.11–0.18 s (room temperature), and possibly others even faster. Whether this time constant, if existent and of the correct value, corresponds to the deactivation of Gα_q_, Gα_11_ and Gα_14_ under normal GAP activity is not known. Interestingly, however, this value is rather similar to that found in mouse rod phototransduction, where the deactivation of rod transducin has been measured to be normally around 0.2 s, although this measurement was made at 37 °C^26^.

Finally, the question remains, what physiological steps do the time constants τ_1_ and τ_2_ in the mathematical fittings of the dim-flash response waveform correspond to? In rod photoreceptors, owing to the rapid gating of the cyclic-nucleotide-gated (CNG) channels^49^, the CNG current provides a reasonably faithful readout of the kinetics of the phototransduction steps upstream. This does not appear to be the case for ipRGCs. Recordings from *Trpc6*^*−/−*^ and *Trpc7*^*−/−*^ single-knockout M1-ipRGCs have revealed subtle changes in the waveform of the dim-flash response (see Figure 5d in Xue et al.^11^). In other words, the light-response kinetics in M1-ipRGCs (possibly both τ_1_ and τ_2_) appear to be dominated by the channel-subunit composition, thus likely the gating of TRPC6,7 ion channels, which is slow^50^.

## Methods

### Animals

All animal experiments were conducted according to the protocols approved by the Institutional Animal Care and Use Committee at Johns Hopkins University. We used the C57BL/6 J strain for control *WT* mice. β-arrestin (arrestin 2 and 3)-single knockouts and the *Grk3*^*−/−*^ mice were made by Dr. Robert Lefkowitz (Duke University). The *β-arrestin1,2*^*fl/fl*^ mice were made by Dr. Gang Pei (Tongji University, CHINA)^51^. Arrestin 1 and 4 knockouts were made by Dr. Cheryl Craft (University of Southern California). The *Grk2*^*fl/fl*^ mice were made by Dr. Gerald Dorn (Washington University in St. Louis). The *Gnb5*^*−/−*^ mice were made by Dr. Ching-Kang Jason Chen (Baylor College of Medicine). The *Rgs2*^*−/−*^ mice were made by Dr. Josef Penninger (University of British Columbia). Mice were raised under 14 h: 10 h light–dark cycle but dark-adapted overnight prior to electrophysiological recordings. Both male and female mice at 1–3 months old were used for experiments. All methods were performed in accordance with the relevant guidelines and regulations, and reported in accordance with ARRIVE guidelines.

### Generation of point mutation knock-in mice

*Opn4*^*6A*^, *Opn4*^*9A*^, and *Plcb4*^*N256A*^ (short for *Opn4*^*6A/6A*^, *Opn4*^*9A/9A*^, and *Plcb4*^*N256A/N256A*^) mice were generated with the CRISPR/Cas9 genome-editing system. The guide sequences and HDR (homology-directed repair) template sequences are summarized in the table below.

**Table:** 

	Guide (5′ to 3′)	HDR template (5′ to 3′)
*Opn4*^*6A*^	GCT GCT CAA TGT GGA GCG GT crRNA made by in-vitro transcription (T7 Quick High Yield RNA Synthesis Kit), using pX330-U6-Chimeric_BB-CBh-hSpCas9 vector	GCC CAG CAC CTG CCT TGC CTT GGG GTG CTT CTC GGT GTA TCA GGC CAG CGC AGC CAC CCC TCC CTC AGC TAC CGC TCT ACC CAC CGC GCG GCA TTG GCT GCG CAG GCC GCG GAC CTC AGC TGG ATC TCT GGA CGG AAG CGT CAA GAG TCC CTG GGT TCT GAG AGT GAA GTG GTA AGT GCC CCC ATT GGC TGG GAC CTC ssODN synthesized by Integrated DNA Technologies (IDT)
*Opn4*^*9A*^	GCT GCT CAA TGT GGA GCG GT crRNA synthesized by Dharmacon	GCC TTG GGG TGC TTC TCG GTG TAT CAG GCC AGC GCA GCC ACC CCT CCC TCG CAT ACC GCG CAG CTC ACC GCG CGG CAT TGG CTG CGC AGG CCG CGG ACC TCA GCT GGA TCT CTG GAC GGA AGC GTC AAG AGT CCC TGG GTT CTG A ssODN synthesized by Dharmacon
*Plcb4*^*N256A*^	CACAGCATCAGCGAGATCCT or AATAAAATTTCATTCAGCCG crRNA synthesized by IDT	AT TTC AAT GAT CTG CAT TGC TCT TTT AGC ATC ATA AAA TGG GAA TAA AAT TTC CGC CAG GCG AGG ATC CCG CTG ATG CTG TGA TGA CAA AAT AAG TCC ATT TAG ATC TAT AAC CCT CAT ACT TGA AAC ssODN synthesized by IDT

Both crRNA and ssODNs were submitted to the JHU Transgenic Facility for pronuclear injections of zygotes from B6SJLF1/J parents (The Jackson Laboratory), together with tracrRNA (Dharmacon) and Cas9 mRNA. Pups with the desired edits were identified by PCR on tail DNA and double-checked by sequencing.

#### ***Opn4***^***6A***^ and ***Opn4***^***9A***^ mice

For *Opn4*^*6A*^, the primer pair Opn4Ala-F (5′- TGT CTG AGC CCA TCA CCA GTG T -3′) and Opn4Ala-R (5′- ACC ACC TCC TCT CTG TCC TGG -3′) were used to amplify a 480-bp PCR product from tail DNA. Digestion of the PCR products with SacII gives a 480-bp band for the wildtype allele and a 240-bp band for the *Opn4*^*6A*^ mutant allele. Founder mice with the correct *Opn4*^*6A*^ allele were bred to *Opn4*^*−/−*^*;Opn4-tdTomato* mice. Progenies positive for *Opn4*^*6A*^ in genotyping were *Opn4*^*6A/-*^. Homozygous *Opn4*^*6A*^ mice were obtained by crossing *Opn4*^*6A/-*^ males to *Opn4*^*6A/-*^ females and selecting for pups negative for the Opn4-knockout allele. *Opn4*^*6A/-*^ and homozygous *Opn4*^*6A*^ mice carrying the *Opn4-tdTomato* BAC transgene (tdTomato label is necessary for identifying ipRGCs) were used for electrophysiological recordings. This breeding scheme was necessary because the presence of the *Opn4-tdTomato* BAC transgene, which contains the wildtype *Opn4* genomic sequence, can interfere with the detection of homozygous *Opn4*^*6A*^ mice. Specifically, wildtype band can be amplified in PCR genotyping with tail samples from *Opn4*^*6A/6A*^*;Opn4-tdTomato* mice. Therefore, we could only be certain of the homozygous status of *Opn4*^*6A*^ in a pup, when both parents of the pup were *Opn4*^*6A/-*^ and the pup doesn’t contain Opn4-knockout allele. Genotyping and breeding of the *Opn4*^*9A*^ mice were performed using the same primer pair and following the same procedure as the *Opn4*^*6A*^ mice.

#### ***Plcb4***^***N256A***^ (short for homozygous ***Plcb4***^***N256A/N256A***^) mice

The primer pair PLCB4F (5’-GTG TCC CTC AGC ATT TAA ACA G-3’) and PLCB4R (5’-AGT AGT GTA CTT CAG ACA GTG TG-3’) were used to amplify a 575-bp PCR from tail DNA. Digestion of the PCR products with BamHI gives a 575-bp band for the wildtype allele, and two bands of 261 bp and 314 bp for the *Plcb4*^*N256A*^ mutant allele.

### Electrophysiology

#### Solutions

For perforated-patch recordings, the base internal solution contained (in mM): 110 KCl, 13NaCl, 2 MgCl_2_, 1 CaCl_2_, 10 EGTA, 10 HEPES, 5 QX314 (pH adjusted to 7.2 with KOH). Amphotericin B was dissolved in DMSO to make 100X stock solution (12.5 mM). The 100X Amphotericin B stock was aliquoted and stored in the dark at − 20 °C for up to two weeks. The complete internal solution was prepared fresh daily by adding 5-μl 100X Amphotericin B stock into 500-μl base internal solution, and sonicated before loading into patch pipette. During recording, retina was perfused with Ames’ medium bubbled with 95%O_2_/5%CO_2_. Synaptic transmission was blocked by adding to the bath 3-mM kynurenic acid, 100-μM hexamethonium bromide, 100-μM picrotoxin and 1-μM strychnine.

#### Tissue preparation

Mice were dark-adapted overnight (typically starting at 18:00, with experiments beginning around noon on the following day), anesthetized by ketamine/xylazine and enucleated before euthanasia in a dark room with minimal red-light illumination. An eye was hemisected and the retina was removed from the eyecup. After removing vitreous with fine forceps, the retina was cut into 4 pieces and stored in bath solution (Ames’ medium + synaptic blockers) bubbled with 95% O_2_/5% CO_2_. When used, a retinal piece was held in the recording chamber, photoreceptor-side down, by a platinum-iridium frame strung with nylon fibers. A patch pipette was used to tear a small hole in the inner limiting membrane to expose a targeted cell. In some cases, another ~ 1-MΩ pipette filled with filtered bath solution was used to further clean the surface of the exposed cell by gentle puffing.

#### Perforated patch recordings of M1-ipRGCs

IpRGCs were genetically labeled by the tdTomato transgene in the BAC transgenic *Opn4-tdTomato* line. M1-ipRGCs were identified by their bright tdTomato fluorescence, small soma size (~ 10 μm in diameter) and dendrites extending into the inner plexiform layer. TdTomato signal was imaged by green (531 nm/40 nm, center wavelength/bandwidth) mercury or xenon arc epifluorescence excitation. In experiments on AAV-injected retinas, GFP signal indicating AAV-infected cell was imaged by blue (440 nm/40 nm, center wavelength/bandwidth) mercury or xenon arc fluorescence excitation. The total duration of epi-illumination was typically 1–10 s. After an M1-ipRGC was identified by epifluorescence, we used infrared optics to establish the patch recording. A period of darkness lasting ~ 30 min separated epifluorescence viewing and the initiation of patch-clamp recording. This dark-adaptation period was extended to 75–90 min for M1-ipRGCs of the *Opn4-Cre;βArr1,2*^*fl/fl*^ or *Opn4*^*9A*^ genotypes, and *Opn4*^*−/−*^ M1-ipRGCs expressing Opn4S^PN^ (short-isoform phospho-null mutant melanopsin) via AAV infection. Patch pipettes were 5–7 MΩ in resistance. A Multiclamp 700B amplifier (Molecular Devices) or an Axopatch 200B amplifier (Axon Instruments) was used. Since rupturing of the patch and transition to whole-cell recording typically leads to run-down of intrinsic photocurrent on M1-ipRGCs, we included QX-314 (a membrane-impermeant blocker of voltage-gated sodium channels) in the internal solution and periodically checked the presence of voltage-gated sodium current to monitor the stability of perforated patch recordings. Liquid-junction potential was corrected. During data acquisition, signal was low-pass filtered at 2 kHz (or 10 kHz) and sampled at 10 kHz (or 50 kHz). Experiments were carried out at room temperature, ~ 23 °C, for better stability.

During data analysis, the dim-flash responses were further low-pass filtered at 2 Hz in software (Clampfit 10.6, Molecular Devices) for fluctuation analysis or kinetics analysis. Responses to bright flashes were filtered at 20 Hz in software when extracting their peak amplitudes and plotting them in figures. Because M1-ipRGCs have slow light response kinetics, the filtering procedure allowed us to get rid of high-frequency noise without distorting the response waveform.

#### Light stimulation

The light source was a 75-W xenon arc lamp, modulated by a heat filter and arrays of calibrated neutral-density and 10-nm-band-pass interference filters. The beam was delivered to the microscope via a fiber-optic light guide, with a shutter in the light path controlled by digital output from the data acquisition program. The size of the light spot was controlled by the field iris diaphragm in the microscope. In most experiments, cells were stimulated with “diffuse light”, with the iris being wide open. When the iris was fully constricted, the light spot had a diameter of 40 μm. Flashes were delivered every 70–120 s (depending on the intensity of the stimulus), sufficient for full recovery after each stimulus. The absolute light intensity was periodically calibrated with a radiometer. The intensity of white light was converted to equivalent 480-nm photons by response-matching in the linear range.

### Virus generation

The WT mouse *Opn4S* and *Opn4L* cDNAs were cloned from mouse retina by RT-PCR into pGEM-T-Easy vector. A plasmid containing the mouse cDNA for OPN4L with 37 out of the 38 C-terminus Serine/Threonine residues mutated to Alanine (Ser376-Thr517) was obtained from Dr. Tian Xue at University of Science and Technology of China. After transferring this cDNA sequence into pGEM-T-Easy vector, site-directed mutagenesis was performed to change Ser372 into alanine, generating cDNA for the phospho-null mutant of *Opn4L* (*Opn4L*^*PN*^). OPN4S and OPN4L differ at their C-terminus tails from Gln454 on. To make cDNA for the phospho-null.

mutant of OPN4S (OPN4S^PN^), a silent mutation (from CCC to CCT, for Pro453) was introduced into OPN4L^PN^ by site-directed mutagenesis to generate a Bsu36I site. The *pGEM-T-Opn4L*^*PN*^ plasmid was then digested with Bsu36I and SpeI (RE site on *pGEM-T* backbone) to remove the coding sequence from Gln454 to stop codon, and replace it with the corresponding part in *Opn4S*^*PN*^ (made by annealing a pair of synthesized DNA oligonucleotides ordered from IDT). The *Opn4S*, *Opn4L*, *Opn4S*^*PN*^ cDNAs were then transferred into the *pAAV-CMV-Luc-IRES-EGFP-SV40* backbone (P1888 from Penn Vector Core, University of Pennsylvania) by restriction enzyme cloning (NheI and BamHI) to replace the luciferase gene (Luc). AAV2 made from the *pAAV-CMV-Opn4-IRES-GFP* vectors resulted in very low Opn4 expression in M1-ipRGCs and were not used for further experiments. *Opn4S-IRES*, *Opn4L-IRES*, *Opn4S*^*PN*^*-IRES* sequences were transferred into the *pAAV-hSyn-EGFP-WPRE-bGH* plasmid (P1696 from Penn Vector Core) by restriction enzyme cloning (EcoRI and NcoI). All constructs were confirmed by sequencing. The *pAAV-hSyn-Opn4-IRES-EGFP-WPRE-bGH* plasmids were sent to Penn Vector Core for production of viral particles to be used in intravireal injection.

### Intravitreal injection

Mice were anesthetized by intraperitoneal injections of Ketamine/Xylazine and placed under a stereomicroscope. A 30G hypodermic needle was first used to create a small hole in the sclera for facilitating the penetration of the glass pipette for injection (pulled from micro-capillaries; Harvard Apparatus, GC150F-10). ~ 1-μL AAV solution per eyeball was pushed into the vitreous chamber of the eye by pressure pulses (~ 12 psi for ~ 200 ms) administered by a Picospritzer. Mice recovered from injections under a heating lamp until they woke up from anesthesia.

### Experimental design and data analysis

For comparing between M1-ipRGCs of different genotypes, each group included four to twenty-nine cells from at least two mice of either sex. For any given cell, to eliminate trial-to-trial variations, we first average at least 5 responses to repeated dim-flash of the same intensity to obtain the ‘averaged response’, which we take to represent the dim-flash-response waveform of the individual cell. Considering that within each genotype there are also cell-to-cell variations in the response waveform, we then take the average of the ‘averaged responses’ obtained from multiple cells of the same genotype. The resulting grand average trace (black traces in Figs. 2 and 4, 5, 6, 7) then provides a general picture of what a typical dim-flash response looks like for a given genotype. Data are all expressed as mean ± SD. Statistical analysis was performed in R. Two-tailed non-parametric tests (Wilcoxon rank sum exact test for two-group comparisons, and Kruskal–Wallis rank sum test for comparisons between more than two groups) were used to determine statistical significance. The Benjamini–Hochberg Procedure was used for multiple testing correction when performing pair-wise comparisons between multiple groups. In all cases, *p* < 0.05 was considered significant.

## Supplementary Information

Supplementary Figures.
