# Supplementary material for: Molecular determinants of response kinetics of mouse M1 intrinsically-photosensitive retinal ganglion cells
Source: Sci Rep. 2021 Dec 6;11:23424. doi: 10.1038/s41598-021-02832-9 (PMC8648817; doi:10.1038/s41598-021-02832-9)
Supplement: Supplementary file 1 — Supplementary Figures. [file 41598_2021_2832_MOESM1_ESM.docx]

**Figure S1 Using *AAV-hSyn-Opn4-IRES-GFP-WPRE* virus to express melanopsin variants in *Opn4^-/-^* M1-ipRGCs.**


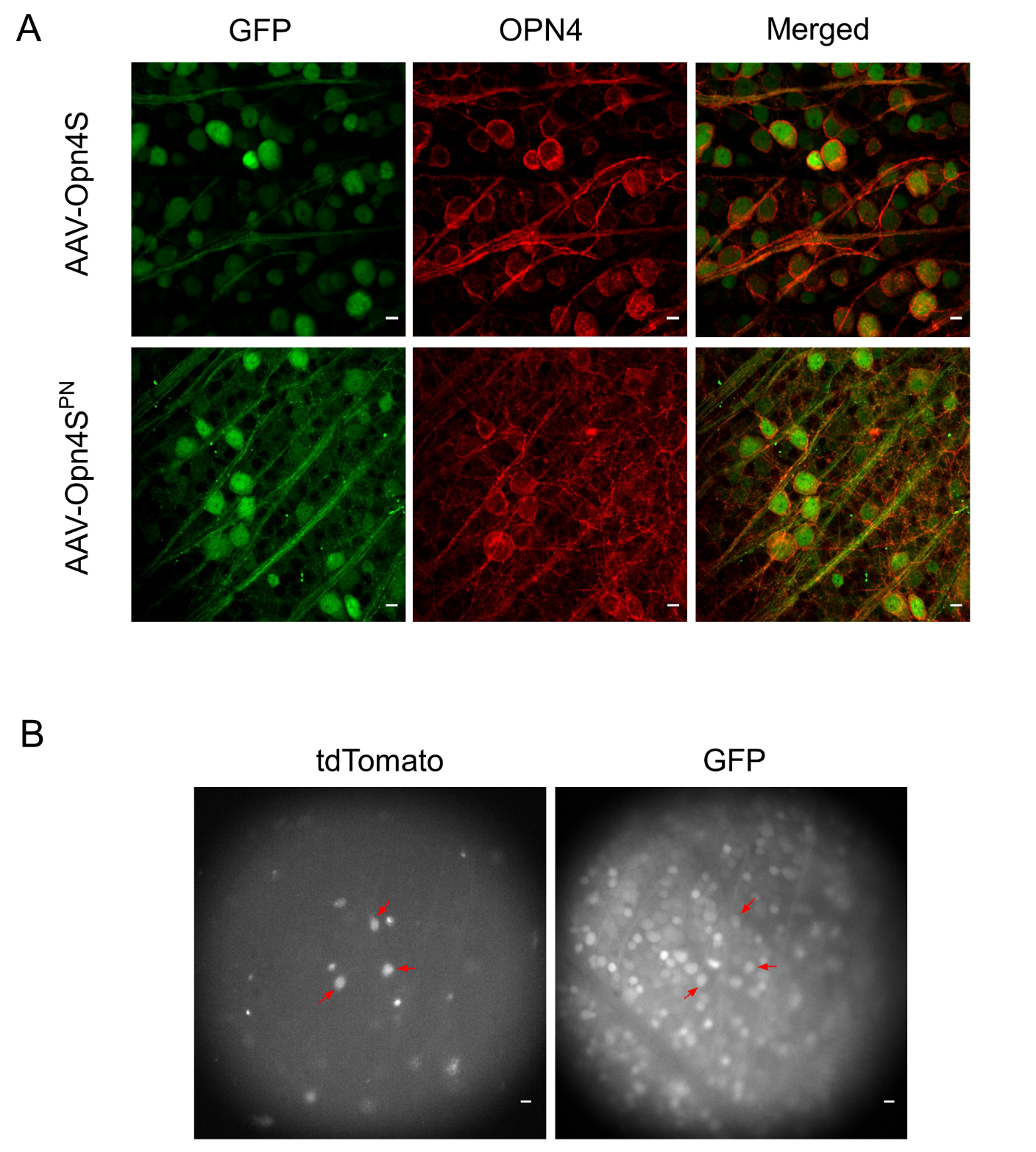


(A) Melanopsin (OPN4) and GFP are co-expressed in *Opn4^-/-^* retinal ganglion cells infected with *AAV-hSyn-Opn4-IRES-GFP-WPRE* virus. Scale bars are 10 μm.

(B) *Opn4^-/-^* M1-ipRGCs infected with *AAV-hSyn-Opn4-IRES-GFP-WPRE* virus (red arrows) were targeted for recording based on colocalization of tdTomato and GFP fluorescence. Scale bars are 10 μm.

**Figure S2 Comparison of intrinsic dim-flash-response parameters in WT vs. *Plcb4^N256A^* M1-ipRGCs.**


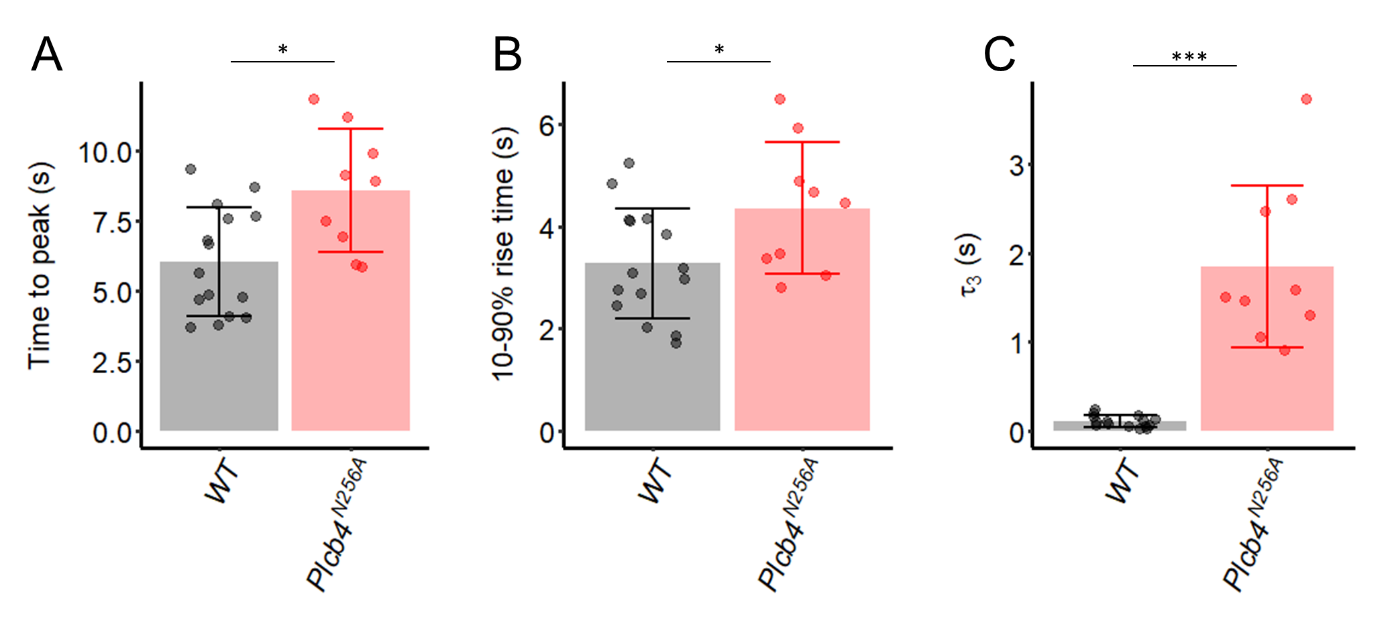


Bars and error bars represent mean ± SD. (*, *p* < 0.05; ***, *p* < 0.001. )

(A) Time to peak: 6.0 ± 1.9 s for WT and 8.6 ± 2.2 s for *Plcb4^N256A^* (*p* = 0.012, Wilcox test).

(B) 10-90% rise time, defined as the time lapse between 10% and 90% of the peak amplitude in the rising phase of the dim-flash response: 3.3 ± 1.1 s for WT and 4.3 ± 1.3 s for *Plcb4^N256A^* (*p* = 0.048, Wilcox test).

(C) τ_3_, defined as the shortest time constant obtained from fitting dim-flash responses from individual cells to $e^{{-t}/{\tau_{1}}}*e^{{-t}/{\tau_{2}}}*e^{{-t}/{\tau_{3}}}$: 0.11 ± 0.066 s for WT and 1.9 ± 0.91 s for *Plcb4^N256A^* (*p* = 1.5×10^-6^, Wilcox test).
